# A cross-sectional multicenter linkage study of hospital admissions and mortality due to methanol poisoning in Iranian adults during the COVID-19 pandemic

**DOI:** 10.1038/s41598-022-14007-1

**Published:** 2022-06-13

**Authors:** Seyed Amirhosein Mahdavi, Nasim Zamani, Rebecca McDonald, Maryam Akhgari, Ali-Asghar Kolahi, Farzad Gheshlaghi, Ali Ostadi, Ahmad Dehghan, Mohammad Moshiri, Morteza Rahbar-Taramsari, Mohammad Delirrad, Neda Mohtasham, Saeed Afzali, Sara Ebrahimi, Pardis Ziaeefar, Navid Khosravi, Amir Mohammad Kazemifar, Mohammadreza Ghadirzadeh, Hoorvash Farajidana, Tahereh Barghemadi, Farangis Sadeghi, Seyed Kaveh Hadeiy, Mehdi Hadipourzadeh, Javad Mesbahi, Mohammad-Reza Malekpour, Mohsen Arabi, Farkhondeh Jamshidi, Bita Dadpour, Knut Erik Hovda, Hossein Hassanian-Moghaddam

**Affiliations:** 1 Legal Medicine Research Center, Legal Medicine Organization, Tehran, Iran; 2 Social Determinants of Health Research Center, Shahid Beheshti University of Medical Sciences, Tehran, Iran; 3 Department of Clinical Toxicology, Loghman-Hakim Hospital, Shahid Beheshti University of Medical Sciences, Tehran, Iran; 4 SERAF, Norwegian Centre for Addiction Research, University of Oslo, Oslo, Norway; 5 Department of Clinical Toxicology, Isfahan Clinical Toxicology Research Center, School of Medicine, Isfahan University of Medical Sciences, Isfahan, Iran; 6 Department of Internal Medicine, Sina Medical Research & Training Hospital, School of Medicine, Tabriz University of Medical Sciences, Tabriz, Iran; 7 Accident Prevention and Crisis Research Center, Shahid Sadoughi University of Medical Sciences and Health Services, Yazd, Iran; 8 Medical Toxicology Research Center, Mashhad University of Medical Sciences, Mashhad, Iran; 9 Department of Forensic Medicine, School of Medicine, Guilan University of Medical Sciences, Rasht, Iran; 10 Department of Forensic Medicine, School of Medicine, Food and Beverages Safety Research Center, Imam Khomeini Hospital, Urmia University of Medical Sciences, Urmia, Iran; 11 Department of Pediatric Clinical Toxicology, Abuzar’s Children Medical Center, Ahvaz Jundishapur University of Medical Sciences, Ahvaz, Iran; 12 Department of Forensic Medicine, School of Medicine, Hamadan University of Medical Sciences, Hamadan, Iran; 13 School of Medicine, Shahid Beheshti University of Medical Sciences, Tehran, Iran; 14 Department of Emergency Medicine, School of Medicine, Mazandaran University of Medical Sciences, Sari, Iran; 15 Department of Internal Medicine, Qazvin University of Medical Sciences, Qazvin, Iran; 16 Department of Emergency Medicine, School of Medicine, Alborz University of Medical Sciences, Karaj, Iran; 17 Clinical Development Research Unit, Seyad Shirazi Hospital, Golestan University of Medial Sciences, Gorgan, Iran; 18 Qom University of Medial Sciences, Qom, Iran; 19 Department of Family Medicine, Sari Medical School, Mazandaran University of Medical Sciences, Sari, Iran; 20 Department of Forensic Medicine and Social Determinant of Health Research Center, Ahvaz Jundishapur University of Medical Sciences, Ahvaz, Iran; 21 The Norwegian CBRNE Centre of Medicine, Department of Acute Medicine, Oslo University Hospital, Oslo, Norway; 22 Loghman-Hakim Hospital Poison Center, South Karegar Street, Kamali St, Tehran, Iran

**Keywords:** Diseases, Eye diseases, Metabolic disorders, Diagnosis, Health policy, Prognosis, Public health

## Abstract

A methanol poisoning outbreak occurred in Iran during the initial months of coronavirus disease 2019 (COVID-19) pandemic. We aimed to evaluate the epidemiology of the outbreak in terms of hospitalizations and deaths. A cross-sectional linkage study was conducted based on the hospitalization data collected from thirteen referral toxicology centers throughout Iran as well as mortality data obtained from the Iranian Legal Medicine Organization (LMO). Patient data were extracted for all cases aged > 19 years with toxic alcohol poisoning during the study period from February until June 2020. A total of 795 patients were hospitalized due to methanol poisoning, of whom 84 died. Median [interquartile ratio; IQR] age was 32 [26, 40] years (range 19–91 years). Patients had generally ingested alcohol for recreational motives (653, 82.1%) while 3.1% (n = 25) had consumed alcohol-based hand sanitizers to prevent or cure COVID-19 infection. Age was significantly lower in survivors than in non-survivors (P < 0.001) and in patients without sequelae vs. with sequelae (P = 0.026). Twenty non-survivors presented with a Glasgow Coma Scale (GCS) score > 8, six of whom were completely alert on presentation to the emergency departments. The time from alcohol ingestion to hospital admission was not significantly different between provinces. In East Azerbaijan province, where hemodialysis was started within on average 60 min of admission, the rate of sequelae was 11.4% (compared to 19.6% average of other provinces)—equivalent to a reduction of the odds of sequelae by 2.1 times [95% CI 1.2, 3.7; p = 0.009]. Older patients were more prone to fatal outcome and sequelae, including visual disturbances. Early arrival at the hospital can facilitate timely diagnosis and treatment and may reduce long-term morbidity from methanol poisoning. Our data thus suggest the importance of raising public awareness of the risks and early symptoms of methanol intoxication.

## Introduction

Iran was the epicenter of the early COVID-19 pandemic in the Middle East, with 1,194,963 cases and 54,574 deaths reported by January 17th 2021^1^. In the absence of effective treatments for the infection, the World Health Organization (WHO) and the United States (US) Centers for Disease Control and Prevention (CDC)^2,3^ recommended wearing face masks, social distancing, and hand sanitization using soap or alcoholic hand sanitizers as key preventive strategies to contain viral spread^2,3^. According to WHO and CDC guidelines, effective alcohol-based hand sanitizers should contain at least 60% ethanol (ethyl alcohol) or 70% isopropanol (isopropyl alcohol)^4,5^.

In Iran, however, false beliefs about supposed COVID-19-protective effects of the ingestion of herbal products, vitamins, trace elements, spices, opium, disinfectants, and sanitizers were common. The high consumer demand for hand sanitizers led to the production of counterfeit and non-standard alcohol-based sanitizers which could be classified into two groups: hand sanitizers with inappropriate proportion of ethanol (< 60%) and hand sanitizers containing methanol^6^.

When misused, alcohol-based sanitizers may be toxic to human health. Ingestion of low-concentrated hydrogen peroxides may result in gastrointestinal (GI) tract irritation. Isopropyl alcohol consumption may lead to severe respiratory and central nervous system depression, and ethanol toxicity may result in a set of common complications^4,7^. When added to hand sanitizers, the serious toxic effects of methanol can occur through inhalational, oral, or dermal exposure^4,7^.

The increased availability of alcohol through hand sanitizers resulted in a mass methanol poisoning in Iran^8,9^, as all provinces reported methanol-poisoned patients from March 7th to April 8th, 2020. Deaths due to methanol poisoning were reported in 26 out of 31 Iranian provinces^10^. In the single province of Fars alone, 797 cases of methanol poisoning and 97 deaths were reported^11^. The 2020 methanol outbreak in Iran thus exceeds the 2013 outbreak in Libya with 1,066 victims and approximately 100 fatalities^12^, previously considered to be the largest outbreak reported.

In an earlier publication, we reported on the epidemiology of the 2020 methanol outbreak in Iran, including total number of deaths (approx. 800) and cases (> 5,800) nationwide, but did not have access to patient data at the time of reporting^13^.

In order to address this gap of knowledge, we conducted a multicenter linkage study, with the aim to investigate the epidemiology of the 2020 methanol poisoning outbreak based on patient data reported by 13 referral toxicology centers and the Iranian Legal Medicine Organization (LMO).

## Materials and methods

### Design

This cross-sectional linkage study was conducted on data collected from 13 referral toxicology centers in affiliation with eleven academic centers (medical universities), as well as mortality data obtained from the LMO. The latter provided the death toll from toxic alcohol poisonings in Iran during the study period from February until May 2020, i.e. during the first wave of COVID-19.

### Setting

Data collection took place at the following 13 referral toxicology centers distributed throughout 11 of the 31 provinces in Iran: Tehran (Loghman Hakim Hospital), Tabriz (Sina Hospital), Isfahan (Alzahra Hospital), Yazd (Shahid Beheshti and Shah Vali Hospital), Ghaemshahr (Vali-e-Asr Hospital), Urmia (Imam Khomeini Hospital), Mashhad (Imam Reza Hospital), Qazvin (Booali-Sina Hospital), Rasht (Razi Hospital), Hamadan (Be’sat Hospital), and Ahvaz (Imam Hospital).

### Participants

Convenience sampling was used to recruit all patients who met the eligibility criteria, without prior calculation of sample size.

#### Inclusion criteria

All alcohol-poisoned patients older than 19 years with a history of illicit alcoholic beverage/hand sanitizer consumption and manifestations of alcohol intoxication including GI symptoms, visual disturbances (VDs), dyspnea, and central nervous system (CNS) signs/symptoms presenting to one of the participating referral toxicology centers between February 22nd and June 30th 2020 were included. Methanol poisoning was diagnosed based on a serum methanol level of 6.25 mmol/L (20 mg/dL) or higher, or a high clinical suspicion of methanol poisoning based on clinical features and a pH < 7.3 and serum bicarbonate < 20 mmol/L^14^.

#### Exclusion criteria

Patients with signs and symptoms and lab tests purely indicative of ethanol poisoning (inebriation without typical GI, VD, and CNS symptoms of methanol poisoning as well as a normal or near normal blood gas analysis) were considered to be ethanol-intoxicated and excluded because serum ethanol concentration could not be routinely measured in the emergency department setting in any of the involved toxicology centers.

### Treatment protocol

All methanol-poisoned patients supposed to be treated according to existing national- and international guidelines^14,15^. Bicarbonate therapy (to correct metabolic acidosis), ethanol therapy (to prevent metabolism of methanol to toxic byproducts), folinic acid (or folic acid in the absence of folinic acid) administration (to facilitate resolving of metabolic acidosis), and hemodialysis (to eliminate methanol, the toxic byproducts, and further correct the acidosis) are the mainstay of treatment. Fomepizole is approved in Iran, but is not available due to high cost.

### Measures

#### Referral toxicology centers

A questionnaire was filled out for every single patient on admission, recording the following: Patients’ demographic characteristics including age, gender, and city (province) of admission, route of exposure (ingestion, inhalation, dermal), cause of consumption (accidental, recreational, suicidal attempt, prevention of COVID-19 infection), place of purchase (pharmacies, supermarkets, herbal shops, hygiene shops, vendors), history of recreational alcohol consumption (chronic users), time elapsed between consumption and hospital presentation (if available), Glasgow Coma Scale (GCS) on admission, need for intubation and hemodialysis (HD), and final outcome (death, survival with- or without sequalae). The time from alcohol intake to hospital admission and the time from arrival to initiation of treatment (see above) were recorded to study possible correlations to outcomes.

Referral toxicology centers were categorized based of the percentage of mortality and sequelae into low and high mortality/sequela rate to make the comparison of sociodemographic and treatments possible.

#### Legal Medicine Organization (LMO)

Since the LMO is responsible for issuing death certificates in cases of unnatural deaths including deaths due to alcohol intoxication, we matched all fatalities due to methanol poisoning reported by the toxicology centers with the mortality data from the LMO during the same period using the patients’ national identification numbers^16^.

### Data analysis

The data was rechecked by two co-authors (SE and PZ) after the questionnaire had been collected electronically from the referral centers to minimize the missing data. Inappropriate and incomplete data were excluded. Data was analyzed using Statistical Package For Social Sciences (SPSS) software version 26, with a significance level of P < 0.05. Mann–Whitney U test was used for analysis of non-normally distributed quantitative data. Kruskal Wallis test was applied to see the continuous data differences among age groups, provinces, and treatments done. A bivariate regression analysis was applied to assess possible variables which independently affect the main outcomes (i.e. death vs. survival and sequelae vs. no-sequelae). We used ROC curve to define the best cut-off for continuous variables based on sensitivity and specificity. A hazard index heatmap was generated by Excel software to show the most vulnerable age and sex groups in every 100,000 inhabitants in each province. The number of inhabitants was retrieved from the statistical center of Iran (https://www.amar.org.ir/english) and was calculated for the study period based on the growth rate in each province.

### Research ethics

The study was approved by the local ethics committee at Shahid Beheshti University of Medical Sciences (IR.SBMU.RETECH.REC.1399.149). Need for written informed consent from the patients or their relatives was waived by ethics committee at Shahid Beheshti University of Medical Sciences due to the fact that the research presented was purely observational and involved no procedures for which written informed consent is normally required. (no 33127).

### Ethics approval and consent to participate

The study was approved by the Ethics Committee of Shahid Beheshti University of Medical Sciences, Tehran, Iran (IR.SBMU.RETECH.REC.1399.149). All experiments were in accordance with Helsinki declaration.

## Results

### Hospitalizations

During the study period, a total of 795 adult patients were hospitalized in thirteen referral toxicology centers (see Table 1). The median age among patients was 32 years [IQR 26, 40] (range 19–91). Males were more commonly admitted due to alcohol poisoning compared to females (P < 0.001), as 90.3% (718 cases) of the patients were male.

**Table 1:** Methanol-poisoned patients' data during the COVID-19 pandemic in Iran (n = 795).

Variable	Death (n = 84)	Survival* (n = 711)			p-value	
		Total (n = 676)	No sequelae (n = 493)	Sequelae (n = 183)	Survivors Vs. dead	Sequelae vs. no sequalae
Male n (%)	76 (90.5)	642 (90.3 )	441 (89.5)	168 (91.8)	0.958^†^	0.363^†^
Female n (%)	8 (9.5)	69 (9.7)	52 (10.5)	15 (8.2)		
Median [IQR]Age (range)(year)	39 (19–68)	31 (19–91)	30 (25, 38)	33 (24, 40)	< 0.001‡	0.026
Median [IQR] Glasgow Coma Score (GCS) (range)**	5 [3, 8] (3–15)	15 [15, 15] (3–15)	15 [15, 15] (3–15)	5 [15, 15] (3–15)	< 0.001	0.001

*IQR* Interquartile range, *GCS* Glasgow Coma Scale.

*Missing data in some cases for defining the sequelae, as they left hospital against medical advise or transferred to another hospital.

^†^Applying Person's Chi-square.

^‡^Applying Man-Whiteny U test.

**GCS could not be measured due to ongoing resucitation on admission is some cases.

### Deaths

In total, there were 84 fatalities among the 795 hospitalized patients (case-fatality rate of 10.6%) prior to discharge (Table1). All 84 deaths due to methanol poisoning were matched between hospital records and LMO mortality reports using the patients’ national identification number. The non-survivors were significantly older than patients who survived methanol poisoning (39 vs. 31 years, P < 0.001). Among the survivors, those who experienced sequelae were also significantly older that other patients without sequelae (33 vs. 30 years, P = 0.026).

### Variation by age and geography

**Figure 1:**
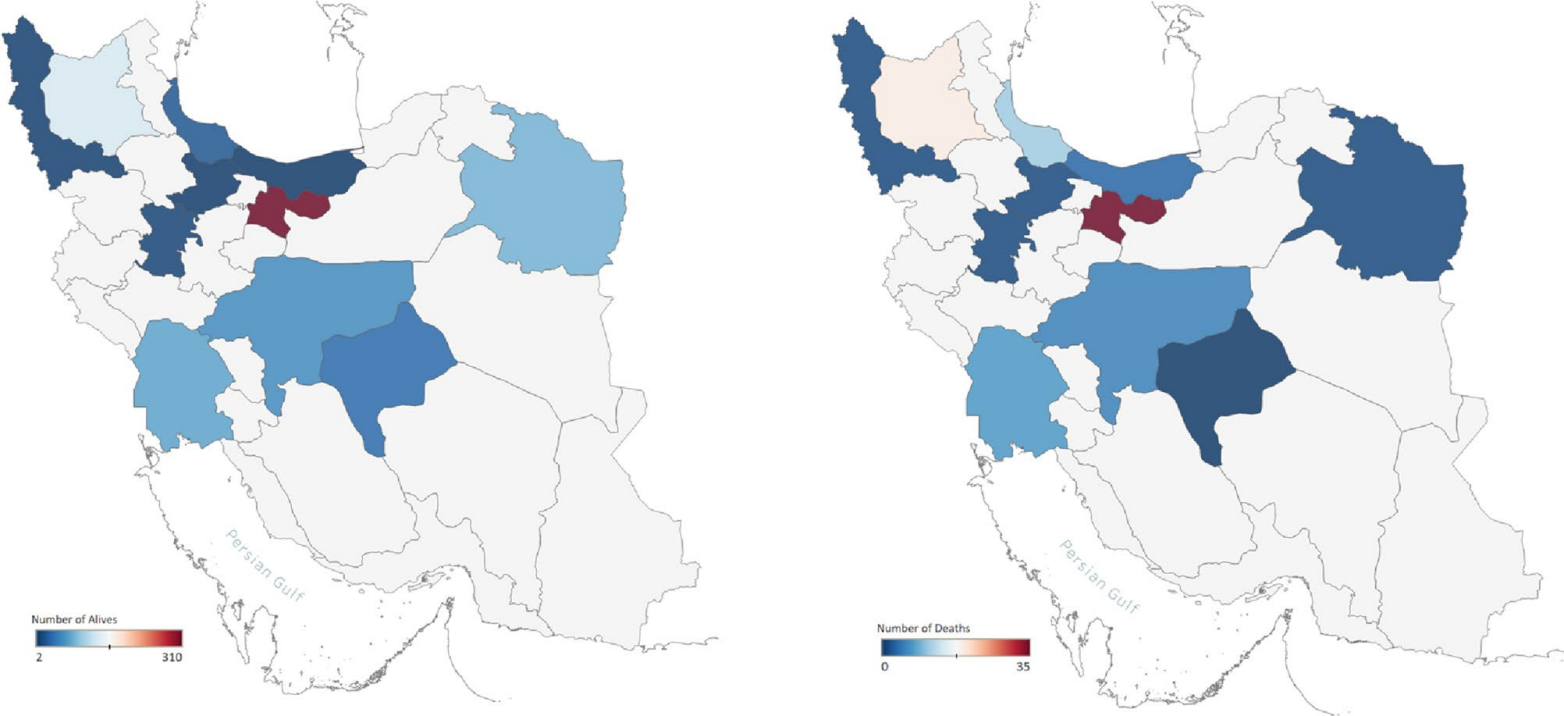
Number of alive (left) and dead (right) patients referred to 11 out of 31 Iranian provinces. Data visualizations were performed using Tableau Desktop, version 2020.1, an interactive data visualization software. (Tableau Software. Seattle, WA; Available through: https://www.tableau.com/).

**Figure 2:**
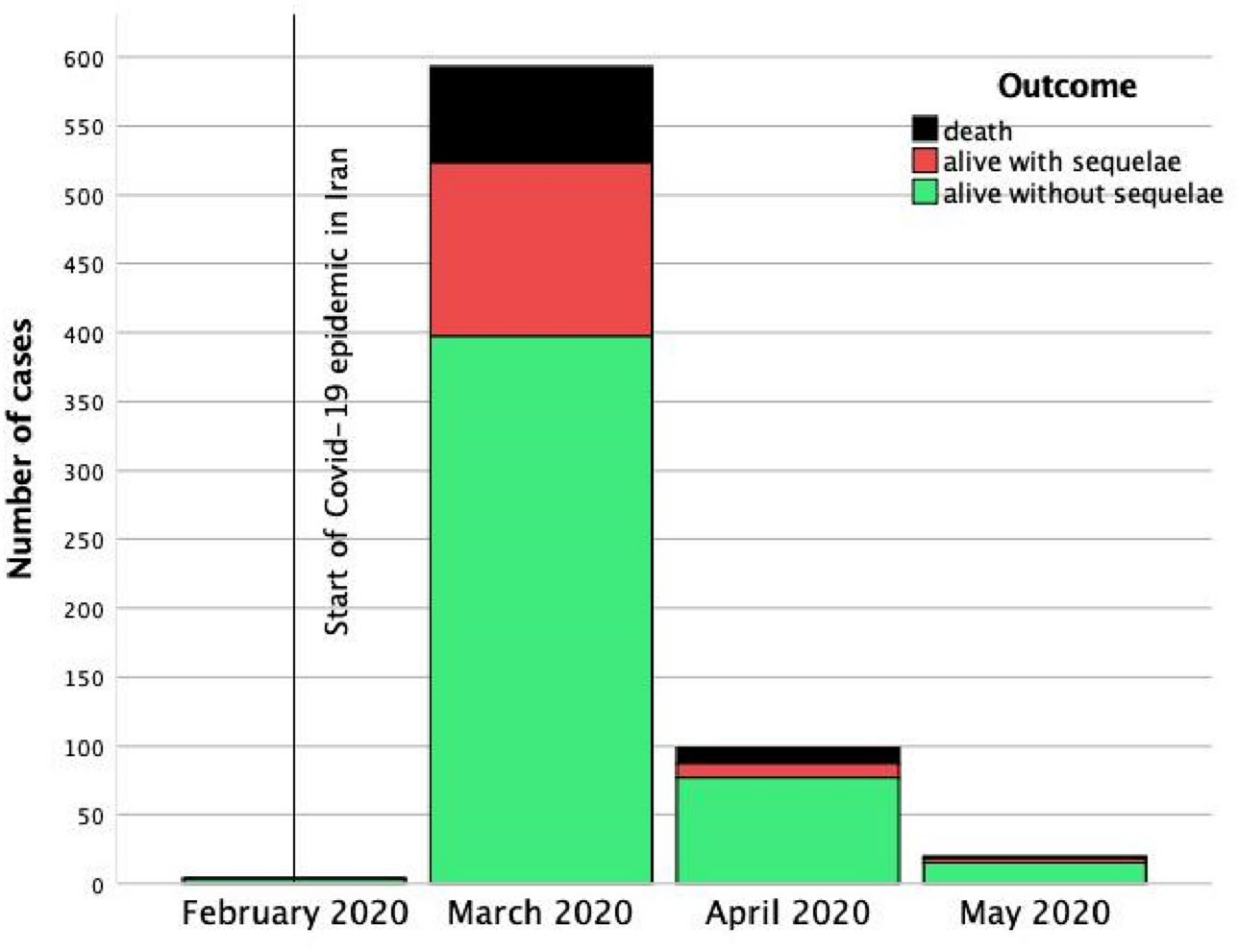
Trend of methanol poisoning after start of Covid-19 in 13 toxicology referral centers.

The number of patients and the mortality rate varied between age groups and the various provinces (Supplementary Table 1, Tables 2 and 3). Overall, 20- to 24-year-old patients had the lowest mortality rate (0.05/100,000), whereas 40- to 44-year-old cases had the highest (0.48/100,000). The capital city (Tehran) had the highest total number of referrals and deaths (Figs. 1 and Supplementary Fig. 1), whereas, after adjustment for population size, Yazd province had the highest rate of referrals among 25- to 30-year-old males (25.49 patients/100,000 population). The highest mortality rate was registered among 40- to 44-year old males in East Azerbaijan with 4.24 deaths/100,000 population. Figure 2 shows the trend of methanol poisoning in the first 4 months following COVID-19 in Iran.

**Table 2:** Deaths per 100,000 population in different age/sex groups in 11 provinces.

Province	Sex	20–24	25–29	30–34	35–39	40–44	45–49	50–54	55–59	60–64	65–69	70–74	75–79	> 80	P	All ages
East Azarbayjan	Female	0.79	0.00	0.00	0.00	0.00	0.00	0.00	0.00	0.00	0.00	0.00	0.00	0.00	0.087	0.05
	Male	0.00	0.00	1.02	1.04	4.24	1.45	3.42	0.00	1.32	0.00	0.00	0.00	0.00		0.88
	P	0.056														
	Both	0.38	0.00	0.52	0.52	2.14	0.73	1.74	0.00	0.64	0.00	0.00	0.00	0.00		0.47
Gilan	Female	0.00	0.00	0.00	0.00	0.00	0.00	0.00	0.00	0.00	0.00	0.00	0.00	0.00	0.063	0.00
	Male	0.00	0.00	1.63	1.64	2.91	0.00	0.00	2.63	3.28	0.00	0.00	0.00	0.00		0.86
	P	0.056														
	Both	0.00	0.00	0.83	0.82	1.44	0.00	0.00	1.30	1.59	0.00	0.00	0.00	0.00		0.43
Hamedan	Female	0.00	0.00	0.00	0.00	0.00	0.00	0.00	0.00	0.00	0.00	0.00	0.00	0.00	0.018	0.00
	Male	0.00	0.00	0.00	0.00	1.45	0.00	0.00	0.00	0.00	0.00	0.00	0.00	0.00		0.11
	P	0.541														
	Both	0.00	0.00	0.00	0.00	0.74	0.00	0.00	0.00	0.00	0.00	0.00	0.00	0.00	0.031	0.06
Isfahan	Female	0.00	0.00	0.00	0.00	0.00	0.00	0.00	0.00	0.00	0.00	0.00	0.00	0.00		0.00
	Male	0.60	0.00	1.07	0.36	0.00	0.00	0.00	0.00	0.00	0.00	0.00	0.00	0.00		0.19
	P	0.210														
	Both	0.30	0.00	0.54	0.18	0.00	0.00	0.00	0.00	0.00	0.00	0.00	0.00	0.00		0.09
Khorasan Razavi	Female	0.00	0.00	0.00	0.00	0.00	0.00	0.00	0.00	0.00	0.00	0.00	0.00	0.00	0.018	0.00
	Male	0.43	0.00	0.00	0.00	0.00	0.00	0.00	0.00	0.00	0.00	0.00	0.00	0.00		0.03
	P	0.541														
	Both	0. 21	0.00	0.00	0.00	0.00	0.00	0.00	0.00	0.00	0.00	0.00	0.00	0.00		0.01
Khuzestan	Female	0.00	0.00	0.39	0.44	0.00	0.00	0.00	0.00	0.00	0.00	0.00	0.00	0.00	0.001	0.08
	Male	0.00	0.00	0.78	0.43	1.14	0.00	0.00	0.00	0.00	0.00	0.00	0.00	0.00		0.20
	P	0.667														
	Both	0.00	0.00	0.59	0.44	0.58	0.00	0.00	0.00	0.00	0.00	0.00	0.00	0.00		0.14
Mazandaran	Female	0.00	0.00	0.00	0.00	0.00	0.00	0.00	0.00	0.00	0.00	0.00	0.00	0.00	0.023	0.00
	Male	0.00	0.00	1.12	0.57	0.00	0.00	0.00	0.00	0.00	0.00	0.00	0.00	0.00		0.18
	P	0.352														
	Both	0.00	0.00	0.57	0.29	0.00	0.00	0.00	0.00	0.00	0.00	0.00	0.00	0.00		0.09
Qazvin	Female	0.00	0.00	0.00	0.00	0.00	0.00	0.00	0.00	0.00	0.00	0.00	0.00	0.00	0.018	0.00
	Male	0.00	0.00	0.00	0.00	0.00	2.17	0.00	0.00	0.00	0.00	0.00	0.00	0.00		0.15
	P	0.541														
	Both	0.00	0.00	0.00	0.00	0.00	1.12	0.00	0.00	0.00	0.00	0.00	0.00	0.00		0.08
Tehran	Female	0.00	0.17	0.00	0.27	0.18	0.22	0.00	0.00	0.00	0.00	0.00	0.00	0.00	0.075	0.07
	Male	0.45	0.18	0.68	1.08	0.68	0.42	0.24	1.45	0.36	0.51	0.00	0.00	0.00		0.43
	P	0.001														
	Both	0.23	0.18	0.34	0.67	0.44	0.32	0.12	0.72	0.18	0.25	0.00	0.00	0.00		0.25
West Azarbayjan	Female	0.00	0.00	0.00	0.00	0.00	0.00	0.00	0.00	0.00	0.00	0.00	0.00	0.00	0.018	0.00
	Male	0.00	0.00	0.00	0.65	0.00	0.00	0.00	0.00	0.00	0.00	0.00	0.00	0.00		0.06
	P	0.541														
	Both	0.00	0.00	0.00	0.33	0.00	0.00	0.00	0.00	0.00	0.00	0.00	0.00	0.00		0.03
Yazd	Female	0.00	0.00	0.00	0.00	0.00	0.00	0.00	0.00	0.00	0.00	0.00	0.00	0.00	1.00	0.00
	Male	0.00	0.00	0.00	0.00	0.00	0.00	0.00	0.00	0.00	0.00	0.00	0.00	0.00		0.00
	P	1.00														
	Both	0.00	0.00	0.00	0.00	0.00	0.00	0.00	0.00	0.00	0.00	0.00	0.00	0.00		0.00
Total	Female	0.06	0.05	0.04	0.13	0.05	0.07	0.00	0.00	0.0	0.00	0.00	0.00	0.00	0.004	0.03
	Male	0.25	0.05	0.64	0.63	0.89	0.31	0.36	0.62	0.45	0.16	0.00	0.00	0.00		0.31
	P	0.514	1.00	0.033	0.020	0.089	0.378	0.319	0.319	0.178	0.514	1.00	1.00	1.00		
	Both	0.16	0.05	0.34	0.39	0.48	0.19	0.18	0.31	0.22	0.08	0.00	0.00	0.00	0.053	0.17

**Table 3:** Median age and time post-ingestion [IQR] distribution of three major outcomes of 13 toxicology referral centers during early Iranian methanol outbreak (n = 716).

Province	East Azarbayjan (n = 140)	Gilan (n = 28)	Hamedan (n = 7)	Isfahan (n = 52)	Khorasan Razavi (n = 56)	Khuzestan (n = 75)	Mazandaran (n = 5)	Qazvin (n = 3)	Tehran (n = 316)	West Azarbayjan (n = 4)	Yazd (n = 30)	P value	Total (n = 716)
**Dead (n = 84)**													
Age (year)	43 [36, 50]	42 [35, 57]	43 [43, 43]	30 [27, 35]	23 [–, –]	36 [30, 40]	32 [32, –]	45 [–, –]	39 [34, 48]	36 [–, –]	–	0.046*	39 [32, 46]
Time of admission post-ingestion (hour)	24 [24, 48]	24 [11, 42]	7 [–, –]	27 [23, 32]	ND	48 [48, 72]	24 [24, –]	24 [–, –]	24 [24, 24]	36 [–, –]	–	0.012^‡^	24 [24, 48]
n (%)	19/140 (13.6)	11/28 (39.3)	1/7 (14.3)	5/52 (9.6)	1/56 (1.8)	7/75 (9.3)	3/5 (60)	1/3 (33.3)	35/316 (11.1)	1/4 (25)	0	< 0.001^†^	84/716 (11.7)
**Survival with no sequelae (n = 492)**													
(Age year)	31 [26, 38]	39 [31, 49]	32 [25, 45]	30 [24, 36]	28 [21, 35]	29 [24, 30]	27 [–, –]	21 [19,–]	32 [26, 43]	27 [27, 27]	26 [24, 30]	< 0.001*	30 [25, 38]
Time of admission post-ingestion (hour)	48 [24, 48]	30 [6, 66]	87 [5, –]	36 [19, 43]	24 [12, 48]	48 [24, 48]	36 [–, –]	48 [48, 48]	24 [24, 24]	24 [36, 48]	21 [12, 24]	0.888^‡^	45 [24, 48]
n (%)	105 (75.0)	10/28 (35.7)	6/7 (85.7)	37 (71.2)	40 (50.6)	66/77 (88)	1/5 (20)	2/3 (66.7)	212/316 (27.1)	1/4 (25)	14/30 (46.7)	< 0.001^†^	492/716 (68.7)
**Survival with sequelae (n = 140)**													
Age (year)	38 [32, 47]	38 [24, 60]	47 [30, –]	33 [24, 39]	31 [25, 37]	41 [28, –]	60 [–, –]	36 [36, 36]	34 [27, 40]	62 [–, –]	26 [24, 33]	0.011*	33 [26, 40]
Time of admission post-ingestion (hour)	24 [24, 48)	72 [20, 96)	0	36 [24, 65)	48 [17, 48)	48 [48, 48)	24 [–, –)	0	48 [24, 48)	13 [–, –)	12 [6, 14)	0.031^‡^	48 [24, 48)
n (%)	16 (11.4)	7/28 (25)	0	10/52 (19.2)	38/56 (67.9)	2/75 (2.7)	1/5 (20)	0	69/316 (21.8)	2/4 (50)	16/30 (53.3)	< 0.001^†^	140/716 (19.6)
P value*													
§	< 0.001	0.750	0.494	0.956	0.150	0.006	0.186	0.259	0.008	0.259	0.625	–	–
^¶^	0.168	0.132	0.999	0.341	0.280	0.760	0.264	0.999	0.001	0.264	0.200	–	0.009
**Total (n = 716)**													
Age (year)	35 [28, 42]	41 [32, 55]	33 [28, 52]	30 [24, 36]	29 [23, 36]	29 [25, 32]	32 [30, 49]	29 [20,43]	34 [28, 42]	47 [33, 60]	26 [25, 30]	< 0.001*	32 [26, 40]
Time of admission post-ingestion (hour)	48 [24, 48]	24 [10, 54]	7 [4, 96]	34 [22, 45]	24 [12, 48]	48 [24, 48]	24 [24, 33]	48 [30, 48]	48 [24, 48]	30 [19, 39]	14 [11, 23]	< 0.001*	40 [24, 48]

*ND* not defined, Missing data treated by random.

*Applying Kruskal Wallis test.

^†^applying Pearson Chi-Square.

^‡^Applying Man-Whitney U-test.

^§^Comparing ages in three outcomes.

^¶^Comparing time of admission post-ingestion.

### Source of exposure

Patients had mostly ingested adulterated alcoholic beverages (92.2%), whereas 7.8% had ingested hand sanitizers and industrial alcohols. Among the 706 (88.8%) cases with complete dataset, the most common cause of ingestion was recreational (82.1%), whereas accidental consumption (3.5%) or intake to prevent COVID-19 (3.1%) were also reported. The cause of alcohol consumption remained undetermined in the remaining 89 cases (11.2%).

Most patients reported having purchased the alcoholic beverages and hand sanitizers from street vendors (465; 58.5%), followed by supermarkets (75; 9.4%), hygiene shops (18; 2.3%), pharmacies (7; 0.9%), and herbal shops (2; 0.3%).

### Time of admission post-ingestion

Among the 684 (86.0%) cases with defined time of exposure, statistical differences were found between fatalities vs. survivors (24 vs. 48 h, P = 0.014, Table 3). The correlation remained significant when comparing fatalities vs. survivors with sequelae (24 vs. 48 h, P = 0.004) as well as fatalities vs. survivors without sequelae (24 vs. 45 h, P = 0.031). After removing the fatalities from the analysis, the time of admission post-ingestion was not different among survivors with sequelae vs. survivors with no sequelae (48 vs. 45 h, P = 0.068). None of the fatalities were admitted sooner than 6 h.

### Clinical symptoms

Among the 338 (42.5%) patient files with complete review, gastro-intestinal symptoms, nausea and/or vomiting (314; 92.89%) were most commonly found. A total of 566/738 patients (76.7%) with complete visual examination had VDs on arrival to the medical care facilities. The examination was not possible in 57 patients who was admitted with loss of consciousness (LOC), unless their family members reported witnessed VDs before LOC of the patient. Five patients became legally blind during the hospital stay, one of whom later died due to complications of methanol toxicity. On presentation, 568 (71.5%) were fully awake with GCS 15/15. Among the fatalities, 20/84 (23.8%) had GCS > 8 on admission. Among these, 6/84 were completely alert (GCS 15) and presented only with GI-symptoms and VDs.

### Treatment

Table 4 shows four different treatments in 716 patients with available data on three major outcomes. There was no significant differences from ingestion to admission time in 13 toxicology centers, while the time of initiation of treatments after arrival were different.

**Table 4:** Median [interquartile range] time of treatments in three major outcomes of 13 toxicology referral centers during early Iranian methanol outbreak.

Time of Treatment (min)	East Azarbayjan (n = 140)	Gilan (n = 28)	Hamedan (n = 7)	Isfahan (n = 52)	Khorasan Razavi (n = 56)	Khuzestan (n = 75)	Mazandaran (n = 5)	Qazvin (n = 3)	Tehran (n = 316)	West Azarbayjan (n = 4)	Yazd (n = 30)Yazd (n = 30)	P value	Total (n = 716)
**Death (n = 84)**													
Buffer													
n (%)	15/19 (79)	1/11 (9)	1/1 (100)	3/5 (60)	0/1 (0)	7/7 (100)	3/3 (100)	1/1 (100)	18/35 (51)	1/1 (100)	0	–	50/84 (60)
Ingestion to tx	1445 [1445, 2885]	2880 [–, –]	450 [–, –]	1500 [1500, –]	–	2885 [2885, 4325]	1480 [1530, –]	1500 [–, –]	1445 [1391, 2885]	4320 [–, –]	–	0.182*	1515 [1445, 2885]
n (%)	18/19 (95)	1/11 (9)	1/1 (100)	4/5 (80)	1/1 (100)	7/7 (100)	3/3 (100)	1/1 (100)	26/35 (74)	1/1 (100)	0	–	62/84 (74)
Arrival to tx	5 [5, 5]	1440 [–, –]	30 [–, –]	150 [75, 225]	5 [–, –]	5 [5, 5]	40 [5, –]	60 [–, –]	5 [5, 128]	2160 [–, –]	–	< 0.001*	5 [5, 60]
Ethanol													
n (%)	15/19 (79)	0/11 (0)	1/1 (100)	4/5 (80)	0/1 (0)	0/7 (0)	3/3 (100)	0/1 (0)	19/35 (54)	0/1(0)	0	–	42/84 (50)
Ingestion to tx	1445 [1445, 2885]	–	440 [–, –]	1665 [1500, 1920]	–	–	1530 [1680, –]	–	1445 [1445, 2885]	–	–	0.282*	1472 [1445, 2345]
n (%)	18 (95)	0/11 (0)	1/1 (100)	5 (100)	1/1 (100)	0/7 (0)	3/3 (100)	0/1 (0)	27/35 (77)	0/1(0)	0	–	54/84 (64)
Arrival to tx	5 [5, 5]	–	20 [–, –]	60 [30, 135]	5 [–, –]	–	5 [90, –]	–	5 [5, 210]	–	–	0.001*	5 [5, 60]
Follinic acid													
n (%)	15/19 (79)	0/11 (0)	1/1 (100)	3/5 (60)	0/1 (0)	6/7 (86)	3/3 (100)	0/1 (0)	16 (46)	1/1 (100)	0	–	45/84 (54)
Ingestion to tx	1445 [1445, 2885]	–	1860 [–, –]	1860 [1440, –]	–	2885 [2885, 4325]	114 [35, –]	–	1472 [1445, 2733]	4440 [–, –]	–	0.242*	1740 [1445, 2885]
n (%)	15/19 (79)	0/11 (0)	1/1 (100)	4/5 (80)	1/1 (100)	6/7 (86)	3/3 (100)	0/1 (0)	23/35 (66)	1/1 (100)	0	–	56/84 (67)
Arrival to tx	5 [5, 5]	–	1440 [–, –]	108 [75, 375]	5 [–, –]	5 [5, 5]	90 [5, –]	–	60 [5, 300]	2280 [–, –]	–	< 0.001*	5 [5, 165]
Hemodialysis													
n (%)	13/19 (68)	1/11 (9)	1/1 (100)	3/5 (60)	0/1 (0)	6/7 (86)	2/3 (67)	1/1 (100)	22/35 (63)	1/1 (100)	0	–	48/84 (57)
Ingestion to tx	1500 [1490, 3060]	1445 [–, –]	1320 [–, –]	1590 [2040,–]	–	3150 [2685, 4515]	1580 [1937, –]	1620 [–, –]	1515 [1486, 2955]	4560 [–, –]	–	0.074*	1605 [1500, 3000]
n (%)	11/19 (58)	1/10 (10)	1/1 (100)	3/5 (60)	1/1 (100)	6/7 (86)	2/3 (67)	1/1 (100)	30 (86)	1/1 (100)	0	–	59/84 (70)
Arrival to tx	60 [60, 135]	60 [–, –]	900 [–, –]	540 [270, –]	600 [–, –]	180 [165, 270]	318 [140, –]	180 [–, –]	60 [60, 128]	2400 [–, –]	–	0.002*	120 [60, 240]
**Survival without sequelae (n = 492)**													
Buffer													
n (%)	93/105 (89)	0/10 (0)	2/6 (33)	33/37 (89)	15 /38 (39)	19/66 (29)	1/1 (100)	0/2 (0)	130/212 (61)	1/1 (100)	0/14 (0)	–	279/492 (55)
Ingestion to tx	2885 [1445, 2885]	–	5415 [390, –]	2220 [1200, 2670]	2885 [1445, 2885]	2885 [1445, 2885]	2990 [–, –]	–	2885 [1445, 2885]	2220 [–, –]	–	0.474*	2885 [1445, 2885]
n (%)	93/105 (90)	0/10 (0)	2/6 (33)	37/37 (100)	25 /38 (66)	19/66 (29)	1/1 (100)	0/2 (0)	131/212 (62)	1/1 (100)	0/14 (0)	–	285/492 (58)
Arrival to tx	5 [5, 5]	–	30 [195, –]	60 [30, 120]	5 [5, 5]	5 [5, 5]	30 [–, –]	–	5 [5, 5]	60 [–, –]	–	< 0.001*	5 [5, 5]
Ethanol													
n (%)	102/105 (97)	0/10 (0)	1/6 (17)	32/37 (86)	16 /38 (42)	0/66 (0)	1/1 (100)	0/2 (0)	165/212 (78)	0/1 (0)	0/14 (0)	–	301/492 (61)
Ingestion to tx	2885 [1445, 2885]	–	390 [–, –]	2235 [1155, 2550]	2885 [1445, 2885]	–	2990 [–, –]	–	2885 [1445, 2885]	–	–	0.094*	2790 [1445, 2885]
n (%)	103/105 (98)	0/10 (0)	1/6 (17)	36/37 (97)	28 /38 (74)	0/66 (0)	1/1 (100)	0/2 (0)	166/212 (78)	0/1 (0)	0/14 (0)	–	307/492 (62)
Arrival to tx	5 [5, 5]	–	30 [–, –]	60 [30, 120]	5 [5, 5]	–	30 [–, –]	–	5 [5, 5]	–	–	< 0.001*	5 [5, 5]
Follinic acid													
n (%)	103/105 (98)	0/10 (0)	0/6 (0)	21/37 (57)	0 /38 (0)	15/66 (23)	1/1 (100)	0/2 (0)	162/212 (76)	1/1 (100)	0/14 (0)	–	303/492 (62)
Ingestion to tx	2885 [1445, 2885]	–	–	1980 [1095, 2850]	2885 [1445, 2885]	2885 [1445, 2885]	6480 [–, –]	–	2885 [1500, 3151]	2220 [–, –]	–	0.092*	2885 [1445, 2885]
n (%)	104/105 (99)	0/10 (0)	0/6 (0)	25/37 (68)	0 /38 (0)	15/66 (23)	1/1 (100)	0/2 (0)	163/212 (77)	1/1 (100)	0/14 (0)	–	309/492 (63)
Arrival to tx	5 [5, 5]	–	–	150 [90, 330]	5 [5, 5]	5 [5, 5]	4320 [–, –]	–	5 [5, 540]	60 [–, –]	–	< 0.001*	5 [5, 5]
Hemodialysis													
n (%)	93/105 (90)	1/10 (10)	1/6 (17)	28/37 (76)	15/38 (39)	16/66 (24)	1/1 (100)	0/2 (0)	185/212 (87)	1/1 (100)	0/14 (0)	–	341/492 (69)
Ingestion to tx	2925 [1530, 2970]	3120 [–, –]	690 [–, –]	2580 [1485, 2940]	3180 [2160, 3600]	3000 [1560, 3210]	2280 [–, –]	–	2220 [1560, 3030]	2760 [–, –]	–	0.072*	2885 [1560, 3000]
n (%)	94/105 (90)	1/10 (10)	1/6 (17)	31/37 (84)	24/38 (63)	16/66 (24)	1/1 (100)	0/2 (0)	186/212 (88)	1/1 (100)	0/14 (0)	–	355/492 (72)
Arrival to tx	60 [49, 90]	240 [–, –]	330 [–, –]	360 [240, 420]	630 [375, 720]	150 [120, 240]	120 [–, –]	–	120 [90, 180]	600 [–, –]	–	< 0.001*	120 [60, 240]
**Survival with sequelae (n = 140)**													
Buffer													
n (%)	15/16 (94)	0/7 (0)	0/7 (0)	7/10 (70)	10/17 (59)	2/2 (100)	1/1 (100)	0	33/69 (48)	1/1 (100)	0/16 (0)	–	59/140 (42)
Ingestion to tx	1445 [1445, 2885]	–	–	2520 [1470, 4320]	2885 [995, 2885]	2885 [2885, 2885]	2220 [–, –]	–	2885 [1500, 3300]	2940 [–, –]		0.181*	2885 [1450, 3060]
n (%)	15/16 (94)	0/7 (0)	0/7 (0)	9/10 (90)	17/17 (100)	2/2 (100)	1/1 (100)	0	35/69 (51)	1/1 (100)	0/16 (0)	–	63/140 (45)
Arrival to tx	5 [5, 5]	–	–	30 [23, 120]	5 [5, 5]	5 [5, 5]	780 [–, –]	–	30 [5, 360]	1500 [–, –]		< 0.001*	5 [5, 82]
Ethanol													
n (%)	16/16 (100)	0/7 (0)	0/7 (0)	7/10 (70)	10/17 (59)	0/2 (0)	0/1 (0)	0	39/69 (57)	1/2 (50)	0/16 (0)	–	63/140 (45)
Ingestion to tx	1445 [1445, 2885]	–	–	2430 [1500, 4320]	2885 [995, 2885]	–	–	–	2885 [1560, 3240]	2940 [–, –]	–	0.039*	2885 [1470, 3060]
n (%)	16/16 (100)	0/7 (0)	0/7 (0)	9/10 (90)	14/17 (82)	0/2 (0)	0/1 (0)	0	39/69 (57)	1/2 (50)	0/16 (0)	–	65/140 (46)
Arrival to tx	5 [5, 5]	–	–	60 [30, 330]	5 [5, 5]	–	–	–	30 [5, 180]	1500 [–, –]	–	< 0.001*	5 [5, 90]
Follinic acid													
n (%)	16/16 (100)	1/7 (43)	1/7 (14)	6/10 (60)	10/17 (59)	2/2 (100)	0/1 (0)	0	39/69 (57)	1/2 (50)	0/16 (0)	–	65/140 (46)
Ingestion to tx	1445 [1445, 2885]	2160 [–, –]	2160 [–, –]	2295 [1500, 3495]	2885 [995, 2885]	2885 [2885, 2885]	–	–	2885 [1560, 3360]	2940 [–, –]	–	0.158*	2885 [1450, 3060]
n (%)	16/16 (100)	1/7 (43)	1/7 (14)	7/10 (70)	16/17 (94)	2/2 (100)	0/1 (0)	0	40/69 (58)	1/2 (50)	0/16 (0)	–	67/140 (48)
Arrival to tx	5 [5, 5]	1080 [–, –]	1080 [–, –]	180 [60, 300]	5 [5, 5]	5 [5, 5]	–		75 [5, 420]	1500 [–, –]	–	< 0.001*	5 [5, 120]
Hemodialysis													
n (%)	14/16 (88)		3/7 (43)	6/10 (60)	10/17 (59)	2/2 (100)	1/1 (100)	0	53/69 (77)	1/2 (50)	0/16 (0)	–	90/140 (64)
Ingestion to tx	1860 [1495, 2948]	3060 [3000–, –]	3000 [3060, –]	2640 [1621, 5325]	3450 [1800, 3765]	3060 [3000,–]	2340 [–, –]	–	2940 [1860, 3270]	3180 [–, –]		0.419*	2885 [1470, 3030]
n (%)	14/16 (88)	1/7 (43)	3/7 (43)	8/10 (80)	16/17 (94)	2/2 (100)	1/1 (100)	0	55/69 (80)	1/2 (50)	0/16 (0)	–	100/140 (71)
Arrival to tx	60 [40, 120]	180 [120, –]	180 [120, –]	240 [195, 690]	660 [420, 885]	180 [120, –]	900 [–, –]	–	120 [30, 300]	1740 [–, –]	–	< 0.001*	120 [60, 420]

*ND* not defined, missing data treated by random.

*Applying Kruskal Wallis test.

^†^applying Pearson Chi–Square.

^‡^Applying Man–Whitney U–test.

Dialysis was performed in 582 (73.2%) of the patients: Among the fatalities, 64/84 (76.2%) had one dialysis-session, and 30/84 (35.7%) had two sessions due to persistent acidosis and/or concomitant VD. Among the survivors, 518/711 (72.9%) had one HD-session, and 109/711 (15.3%) had two sessions (P < 0.001). No other significant differences were detected between the two groups of deaths and survivors. Among the 479 survived cases with documented VDs on arrival and visual examination on discharge, 343 (71.6%) were treated with HD and medical management, and their VD were resolved [OR 55.6 (95%CI, 7.6, 333.3); p < 0.001].

Comparing survivors with and without sequela (n = 632), those provinces (East Azerbaijan) that started hemodialysis within average 60 min post admission (Table 4), the rate of sequelae was 11.4% (compared to 19.6% average of other provinces). This shows odds of 2.1 times less sequelae in East Azerbaijan [95% CI 1.2, 3.7, p = 0.009] compared to other provinces.

### Discharge

Among the survivors, 492/632 (77.8%) had no sequelae on discharge, whereas 133/632 (21.1%) had visual disturbances and 7/632 (1.1%) had neurological complications. The data on health status was not available in 79/795 (9.9%) cases as they left the hospital against medical advice, or were transferred to other hospitals essentially due to lack of ICU beds.

### Bivariate regression analysis

Table 5 displays regression analysis for the two main outcomes of death (alive/dead) and sequelae among survivors (present/not present). For death, a GCS score equal or below 9 points and age > 36 years were independent predictors. A GCS score equal or below 9 points, age > 31 years, and provenance from any province except for East Azerbaijan (with a sequela rate of 11.4% vs. average sequelae of 19.6%) independently predicted presence of sequelae on discharge. Yazd and Khorsan Razavi (with mortality rate of 0.9%) were significantly different from other provinces with average mortality of 10.6% (OR 15.2, 95% CI 2.1, 110.3, p < 0.001) in univariate analysis. However, in regression analysis, the patient’s province of residence did not predict mortality.

**Table 5:** Logistic regression analysis for independent predictive factors of death and sequelae in methanol outbreak based on on-arrival sociodemographic variables.

Variable	Beta	SE	OR (95% CI)	R^#^	P value
Death* (yes vs. no)					
GCS < = 9	4.57	0.66	100.00 (27.03–333.33)	0.622	< 0.001
Age > 36	1.21	0.52	3.33 (1.21, 9.26)		
Sequelae** (yes vs. no)					
GCS < = 9	1.30	0.48	3.31 (1.43, 9.33)	0.193	0.003
Age > 31	0.69	0.25	2.00 (1.22, 3.26)		
Provinces other than East Azerbaijan	0.88	0.35	2.42 (1.22, 4.78)		

^**#**^Nagelkerke R Square.

*All variables with p value less than 0.2 were entered in the model including: ingestion time to admission, age, province mortality rate (high vs. low), schooling categorization (more than 12 years; yes vs. no), Glasgow Coma Scale (GCS).

**All variables with p value less than 0.2 were entered in the model including: ingestion time to admission, age, province sequelae rate (high vs. low), GCS.

## Discussion

In this study, we report on 795 adult patients who were hospitalized for methanol poisoning in thirteen Iranian referral toxicology centers, of whom 84 patients died.

In methanol poisoning, the outcome is typically defined by the level of consciousness on arrival, the severity of the acidosis, and also ability of respiratory compensation of metabolic acidosis. Vice versa, a lack of ability to hyperventilate when acidotic has been shown as a poor prognostic factor^15,17,18^.

Our findings are unexpected in that they show a difference in outcome between the age groups, and suggest that early admission was associated with fatal outcome. Older patients were more prone to late complications and death, possibly due to reluctance to seek medical aid or due to background diseases.

Paradoxically, the fatalities in our study arrived earlier compared to survivors (24 h vs. 45–48 h). Death from methanol poisoning requires metabolism to—and accumulation of—formate, and it takes time to reach fatal levels. A possible explanation is that the dead cases consumed pure methanol to a greater degree, whereas survivors might have ingested a blend of ethanol and methanol (which may have caused them to deteriorate and seek medical care later). However, due to lack of laboratory confirmation of ethanol, methanol, and byproducts, we are unable to test this hypothesis in this study, and any interpretations remain speculative.

The current data shows how methanol poisoning affects patients as young as in their twenties who, if they survive with sequelae, will face many years of disability due to visual impairment (see Table 3). Further, the high fatality rate (approximately 10%) and the fact that six of the fatalities were completely alert on admission, illustrates the difficulties of diagnostic- and therapeutic strategies of methanol poisoning. Limited facilities to treat methanol poisoning during the COVID-19 pandemic may have worsened the situation, and limitations in diagnostic facilities may have further added to the high death toll^8,12^. Moreover, patient fears of COVID-19 infection in the hospital setting may have led to late presentations.

Iran is one of the countries where methanol mass poisonings occur frequently, likely due to limited access to legal alcohol. This has become a major health concern in recent years^14,15^. Alcohol availability in Iran has become further complicated after the price of standard manufactured alcoholic beverages significantly increased due to the economic sanctions against the country introduced by the US administration in 2017^19–22^. Major outbreaks had previously been reported in Iran, e.g. in 2013 and 2018, the latter involving 768 patients and 96 deaths in 21 provinces^21^. In none of the outbreaks before COVID-19, hand sanitizers had any role as a source of methanol poisoning.

Since the beginning of the COVID-19 pandemic, many reports have been published warning about the dangers of methanol content in hand-sanitizers and in bootlegged alcohol sold for consumption in Iran^8,13,22^. However, clinical complications from drinking hand-sanitizers is a global challenge, including Asian countries as well as the US^23^, as false beliefs about preventing COVID-19 infection by ingestion of alcoholic hand sanitizers have claimed numerous human lives^24,25^.

With limited access to standard alcoholic beverages and hand sanitizers, Muslim countries like Iran, Malaysia, and Indonesia are more vulnerable to these sub-standard products^25^. Worldwide reports of methanol outbreaks during the pandemic (https://msf.no/mpi, accessed 29th Sept 2021) should prompt urgent action to address the issue.

On April 5th 2020, a warning was issued by WHO on the hazardous effects of ingestion of bleach and alcoholic hand-sanitizers^26^. More efforts are however warranted to prevent methanol poisoning by ingestion of non-standard alcohols all around the world.

The healthcare burden of methanol poisoning becomes especially apparent during mass poisonings with high volume of patients referring with a possible diagnosis of methanol poisoning. The system may then become inefficient or even collapse^15^. Lack of access to antidotes (ethanol or fomepizole) and other treatment facilities (including hemodialysis and ICU capacities) are other concerns that should be addressed to ensure better preparedness for future events. A stronger focus on “active case finding” through traditional and social media^27^ is likely to prove beneficial in a majority of cases. Treatment should be initiated as early as possible to reduce the risk of sequelae and death.

### Limitations

The reported fatality rates are based on the data recorded in thirteen toxicology referral hospitals that agreed to take part in this study. These patient data are not exhaustive, as the hospitals only represent eleven of the 31 provinces in Iran. As a result, our sample size (n = 795 patients) is only a fraction of the total count of 5,876 hospitalizations due to methanol poisoning during the first quarter of COVID-19 that we reported on in an earlier publication^20^.

Fatalities due to methanol poisoning may have occurred without patient referral to a toxicological referral centers, suggesting that the mortality rate presented in this study is a conservative estimate. Also, some of the methanol intoxicated patients had concomitant COVID-19 infection, and if they died, their bodies were not sent to LMO for autopsy due to infection. Their death certificate would be issued by the health care authorities, and thus not entered into the LMO data.

Lack of analyses for methanol, ethanol and formic acid in the current study may reduce the internal validity of data. Furthermore, the time to admission was based on patient (or family member) self-reporting, and thus likely subject to recall and social desirability biases. Similarly, time to admission does not equal to time to treatment in most of the cases (especially for HD). Finally, possible variations in in the treatment and data collection practices between the eleven provinces mean that direct comparisons may not be feasible.

## Conclusion

Our results suggest that older patients were more prone to fatal outcome, whereas younger patients were more likely to survive. However, in the absence of laboratory data of methanol and ethanol concentrations on admission, this finding needs to be interpreted with great caution. Generally speaking, early arrival at the hospital can facilitate timely diagnosis and treatment and may reduce long-term morbidity from methanol poisoning. Our data thus suggest the importance of raising public awareness of the risks and early symptoms of methanol intoxication.

## Supplementary Information

Supplementary Information.

## Data Availability

The datasets generated and/or analyzed during the current study are available from the corresponding author on reasonable request.

## Abbreviations

ABHRs: Alcohol-based Hand Rubs
CDC: Centers for Disease Control and Prevention
CI: Confidence interval
GCS: Glasgow Coma Scale
HD: Hemodialysis
OR: Odds ration
SPSS: Statistical Package for the Social Sciences
TGA: Therapeutic Goods Administration
VDs: Visual disturbances
WHO: World Health Organization
