# Supplementary material for: A cross-sectional multicenter linkage study of hospital admissions and mortality due to methanol poisoning in Iranian adults during the COVID-19 pandemic
Source: Sci Rep. 2022 Jun 13;12:9741. doi: 10.1038/s41598-022-14007-1 (PMC9189800; doi:10.1038/s41598-022-14007-1)
Supplement: Supplementary file 1 — Supplementary Information. [file 41598_2022_14007_MOESM1_ESM.pdf]

Supplementary Table 1: Survivors per 100,000 population in different age/sex groups in 11 provinces

Province	Sex	20-24	25-29	30-34	35-39	40-44	45-49	50-54	55-59	60-64	65-69	70-74	75-79	>80	P	All ages
East Azarbayjan	Female	1.59	1.30	0.53	1.57	0.00	0.00	0.00	0.00	0.00	0.00	0.00	0.00	0.00	0.001	0.40
	Male	13.24	15.95	10.15	15.54	1.82	5.80	5.98	6.38	2.63	0.00	0.00	3.85	0.00		5.93
	P	0.050														
	Both	7.63	8.83	5.43	8.59	0.92	2.93	3.04	3.19	1.27	0.00	0.00	1.89	0.00		3.21
Gilan	Female	0	1.00	0.00	0.00	0.00	0.00	0.00	0.00	0.00	0.00	0.00	0.00	0.00		0.08
	Male	3.49	0.96	1.63	2.46	0.97	3.16	1.10	1.32	1.64	2.27	3.45	0.00	0.00	<0.001	1.40
	P	0.551														
	Both	1.75	0.98	0.83	0.23	0.48	1.57	0.55	0.65	0.79	1.09	1.64	0.00	0.00		0.74
Hamedan	Female	0.00	0.00	0.00	0.00	1.49	0.00	0.00	0.00	2.86	0.00	0.00	0.00	0.00	0.454	0.23
	Male	1.72	1.32	3.30	0.00	0.00	0.00	0.00	0.00	3.03	0.00	0.00	0.00	0.00		0.67
	P	0.006														
	Both	0.87	0.66	1.66	0.00	0.74	0.00	0.00	0.00	2.94	0.00	0.00	0.00	0.00		0.45
Isfahan	Female	1.82	0.47	0.36	0.74	0.00	0.00	0.00	0.00	0.00	0.00	0.00	0.00	0.00		0.27
	Male	6.55	4.67	3.20	2.55	0.94	2.75	0.62	0.77	0.96	0.00	0.00	0.00	0.00	0.012	1.75
	P	0.008														
	Both	4.20	2.59	1.79	1.66	0.48	1.42	0.32	0.39	0.48	0.00	0.00	0.00	0.00		1.02
Khorasan Razavi	Female	0.85	0.71	0.60	0.32	0.00	0.00	0.00	0.00	0.00	0.00	0.00	0.00	0.00	0.021	0.21
	Male	10.30	4.27	4.99	3.75	0.81	1.54	1.23	0.00	0.92	0.00	0.00	0.00	0.00		2.14
	P	0.005														
	Both	5.57	2.50	2.81	2.05	0.41	0.78	0.62	0.00	0.45	0.00	0.00	0.00	0.00		1.18
Khuzestan	Female	1.14	0.00	0.39	0.00	0.00	0.00	0.00	0.00	0.00	0.00	0.00	0.00	0.00	0.094	0.12
	Male	9.44	9.78	7.39	2.59	1.14	0.00	0.87	0.00	0.00	0.00	0.00	0.00	0.00		2.71
	P	0.006														
	Both	5.35	4.90	3.91	1.31	0.58	0.00	0.44	0.00	0.00	0.00	0.00	0.00	0.00		1.43`
Mazandaran	Female	0.00	0.75	0.00	0.00	0.00	0.00	0.00	0.00	0.00	0.00	0.00	0.00	0.00	0.999	0.06
	Male	0.00	0.00	0.00	0.00	0.00	0.00	0.00	0.00	1.41	0.00	0.00	0.00	0.00		0.06
	P	0.004														
	Both	0.00	0.37	0.00	0.00	0.00	0.00	0.00	0.00	0.68	0.00	0.00	0.00	0.00		0.06
Qazvin	Female	0.00	0.00	0.00	0.00	0.00	0.00	0.00	0.00	0.00	0.00	0.00	0.00	0.00	0.352	0.00
	Male	4.76	0.00	0.00	1.45	0.00	0.00	0.00	0.00	0.00	0.00	0.00	0.00	0.00		0.45
	P	0.023														

	Both	2.35	*0.00*	0.00	*0.75*	0.00	*0.00*	*0.00*	*0.00*	*0.00*	*0.00*	*0.00*	*0.00*	*0.00*		*0.23*
Tehran	Female	2.53	1.40	0.80	0.27	0.71	0.44	0.24	0.00	0.35	0.00	0.00	0.00	0.00	<0.001	0.51
	Male	10.66	8.30	7.40	4.99	4.97	2.95	3.55	3.77	3.64	2.04	3.94	0.00	1.15		3.96
	P	0.165														
	Both	6.63	4.80	4.05	2.62	2.88	1.73	1.90	1.87	1.96	1.00	1.92	0.00	0.56		2.25
West Azerbaijan	Female	0.00	0.00	0.00	0.00	0.00	0.00	0.00	0.00	0.00	0.00	0.00	0.00	0.00	0.114	0.00
	Male	0.00	0.65	0.00	0.65	0.00	0.00	0.00	2.82	0.00	2.78	0.00	0.00	0.00		0.29
	P	0.041														
	Both	0.00	0.33	0.00	0.33	0.00	0.00	0.00	1.41	0.00	1.30	0.00	0.00	0.00		0.15
Yazd	Female	7.50	4.17	0.00	0.00	0.00	0.00	0.00	0.00	0.00	0.00	0.00	0.00	0.00	0.150	0.85
	Male	9.30	25.49	7.94	1.61	4.44	0.00	3.33	0.00	0.00	0.00	0.00	0.00	0.00		4.17
	P	0.002														
	Both	8.43	15.00	4.03	0.84	2.33	0.00	1.75	0.00	0.00	0.00	0.00	0.00	0.00		2.56
Total	Female	1.45	0.85	0.44	0.33	0.27	0.13	0.07	0.00	0.22	0.00	0.00	0.00	0.00	<0.001	0.29
	Male	7.83	6.54	5.15	3.88	2.14	2.08	2.02	2.05	1.89	0.97	1.52	0.37	0.32		2.63
	P	0.006	0.024	0.008	0.002	0.028	0.089	0.010	0.039	0.039	0.178	0.319	0.514	0.514	0.001	
	Both	4.68	3.70	2.81	2.16	1.22	1.12	1.06	1.02	1.04	0.47	0.72	0.18	0.16	0.149	1.47

Alives Per 100,000 (0.00 – 25.49)

Age Group	Sex	East Azarbayjan	Gilan	Hamedan	Isfahan	Khorasan Razavi	Khuzestan	Mazandaran	Qazvin	Tehran	West Azarbayjan	Yazd	All 11 Provinces
20-24	Female	1.59	0.00	0.00	1.82	0.85	1.14	0.00	0.00	2.53	0.00	7.50	1.45
	Male	13.24	3.49	1.72	6.55	10.30	9.44	0.00	4.76	10.66	0.00	9.30	7.83
	Both	7.63	1.75	0.87	4.20	5.57	5.35	0.00	2.35	6.63	0.00	8.43	4.68
25-29	Female	1.30	1.00	0.00	0.47	0.71	0.00	0.75	0.00	1.40	0.00	4.17	0.85
	Male	15.95	0.96	1.32	4.67	4.27	9.78	0.00	0.00	8.30	0.65	25.49	6.54
	Both	8.83	0.98	0.66	2.59	2.50	4.90	0.37	0.00	4.80	0.33	15.00	3.70
30-34	Female	0.53	0.00	0.00	0.36	0.60	0.39	0.00	0.00	0.80	0.00	0.00	0.44
	Male	10.15	1.63	3.30	3.20	4.99	7.39	0.00	0.00	7.40	0.00	7.94	5.15
	Both	5.43	0.83	1.66	1.79	2.81	3.91	0.00	0.00	4.05	0.00	4.03	2.81
35-39	Female	1.57	0.00	0.00	0.74	0.32	0.00	0.00	0.00	0.27	0.00	0.00	0.33
	Male	15.54	2.46	0.00	2.55	3.75	2.59	0.00	1.45	4.99	0.65	1.61	3.88
	Both	8.59	1.23	0.00	1.66	2.05	1.31	0.00	0.75	2.62	0.33	0.84	2.16
40-44	Female	0.00	0.00	1.49	0.00	0.00	0.00	0.00	0.00	0.71	0.00	0.00	0.27
	Male	1.82	0.97	0.00	0.94	0.81	1.14	0.00	0.00	4.97	0.00	4.44	2.14
	Both	0.92	0.48	0.74	0.48	0.41	0.58	0.00	0.00	2.88	0.00	2.33	1.22
45-49	Female	0.00	0.00	0.00	0.00	0.00	0.00	0.00	0.00	0.44	0.00	0.00	0.13
	Male	5.80	3.16	0.00	2.75	1.54	0.00	0.00	0.00	2.95	0.00	0.00	2.08
	Both	2.93	1.57	0.00	1.42	0.78	0.00	0.00	0.00	1.73	0.00	0.00	1.12
50-54	Female	0.00	0.00	0.00	0.00	0.00	0.00	0.00	0.00	0.24	0.00	0.00	0.07
	Male	5.98	1.10	0.00	0.62	1.23	0.87	0.00	0.00	3.55	0.00	3.33	2.02
	Both	3.04	0.55	0.00	0.32	0.62	0.44	0.00	0.00	1.90	0.00	1.75	1.06
55-59	Female	0.00	0.00	0.00	0.00	0.00	0.00	0.00	0.00	0.00	0.00	0.00	0.00
	Male	6.38	1.32	0.00	0.77	0.00	0.00	0.00	0.00	3.77	2.82	0.00	2.05
	Both	3.19	0.65	0.00	0.39	0.00	0.00	0.00	0.00	1.87	1.41	0.00	1.02
60-64	Female	0.00	0.00	2.86	0.00	0.00	0.00	0.00	0.00	0.35	0.00	0.00	0.22
	Male	2.63	1.64	3.03	0.96	0.92	0.00	1.41	0.00	3.64	0.00	0.00	1.89
	Both	1.27	0.79	2.94	0.48	0.45	0.00	0.68	0.00	1.96	0.00	0.00	1.04
65-69	Female	0.00	0.00	0.00	0.00	0.00	0.00	0.00	0.00	0.00	0.00	0.00	0.00
	Male	0.00	2.27	0.00	0.00	0.00	0.00	0.00	0.00	2.04	2.78	0.00	0.97
	Both	0.00	1.09	0.00	0.00	0.00	0.00	0.00	0.00	1.00	1.30	0.00	0.47
70-74	Female	0.00	0.00	0.00	0.00	0.00	0.00	0.00	0.00	0.00	0.00	0.00	0.00
	Male	0.00	3.45	0.00	0.00	0.00	0.00	0.00	0.00	3.94	0.00	0.00	1.52
	Both	0.00	1.64	0.00	0.00	0.00	0.00	0.00	0.00	1.92	0.00	0.00	0.72
75-79	Female	0.00	0.00	0.00	0.00	0.00	0.00	0.00	0.00	0.00	0.00	0.00	0.00
	Male	3.85	0.00	0.00	0.00	0.00	0.00	0.00	0.00	0.00	0.00	0.00	0.37
	Both	1.89	0.00	0.00	0.00	0.00	0.00	0.00	0.00	0.00	0.00	0.00	0.18
80 Plus	Female	0.00	0.00	0.00	0.00	0.00	0.00	0.00	0.00	0.00	0.00	0.00	0.00
	Male	0.00	0.00	0.00	0.00	0.00	0.00	0.00	0.00	1.15	0.00	0.00	0.32
	Both	0.00	0.00	0.00	0.00	0.00	0.00	0.00	0.00	0.56	0.00	0.00	0.16
All Ages	Female	0.40	0.08	0.23	0.27	0.21	0.12	0.06	0.00	0.51	0.00	0.85	0.29
	Male	5.93	1.40	0.67	1.75	2.14	2.71	0.06	0.45	3.96	0.29	4.17	2.63
	Both	3.21	0.74	0.45	1.02	1.18	1.43	0.06	0.23	2.25	0.15	2.56	1.47

Deaths Per 100,000 (0.00 – 4.24)

Age Group	Sex	East Azarbayjan	Gilan	Hamedan	Isfahan	Khorasan Razavi	Khuzestan	Mazandaran	Qazvin	Tehran	West Azarbayjan	Yazd	All 11 Provinces
20-24	Female	0.79	0.00	0.00	0.00	0.00	0.00	0.00	0.00	0.00	0.00	0.00	0.06
	Male	0.00	0.00	0.00	0.60	0.43	0.00	0.00	0.00	0.45	0.00	0.00	0.25
	Both	0.38	0.00	0.00	0.30	0.21	0.00	0.00	0.00	0.23	0.00	0.00	0.16
25-29	Female	0.00	0.00	0.00	0.00	0.00	0.00	0.00	0.00	0.17	0.00	0.00	0.05
	Male	0.00	0.00	0.00	0.00	0.00	0.00	0.00	0.00	0.18	0.00	0.00	0.05
	Both	0.00	0.00	0.00	0.00	0.00	0.00	0.00	0.00	0.18	0.00	0.00	0.05
30-34	Female	0.00	0.00	0.00	0.00	0.00	0.39	0.00	0.00	0.00	0.00	0.00	0.04
	Male	1.02	1.63	0.00	1.07	0.00	0.78	1.12	0.00	0.68	0.00	0.00	0.64
	Both	0.52	0.83	0.00	0.54	0.00	0.59	0.57	0.00	0.34	0.00	0.00	0.34
35-39	Female	0.00	0.00	0.00	0.00	0.00	0.44	0.00	0.00	0.27	0.00	0.00	0.13
	Male	1.04	1.64	0.00	0.36	0.00	0.43	0.57	0.00	1.08	0.65	0.00	0.63
	Both	0.52	0.82	0.00	0.18	0.00	0.44	0.29	0.00	0.67	0.33	0.00	0.39
40-44	Female	0.00	0.00	0.00	0.00	0.00	0.00	0.00	0.00	0.18	0.00	0.00	0.05
	Male	4.24	2.91	1.45	0.00	0.00	1.14	0.00	0.00	0.68	0.00	0.00	0.89
	Both	2.14	1.44	0.74	0.00	0.00	0.58	0.00	0.00	0.44	0.00	0.00	0.48
45-49	Female	0.00	0.00	0.00	0.00	0.00	0.00	0.00	0.00	0.22	0.00	0.00	0.07
	Male	1.45	0.00	0.00	0.00	0.00	0.00	0.00	2.17	0.42	0.00	0.00	0.31
	Both	0.73	0.00	0.00	0.00	0.00	0.00	0.00	1.12	0.32	0.00	0.00	0.19
50-54	Female	0.00	0.00	0.00	0.00	0.00	0.00	0.00	0.00	0.00	0.00	0.00	0.00
	Male	3.42	0.00	0.00	0.00	0.00	0.00	0.00	0.00	0.24	0.00	0.00	0.36
	Both	1.74	0.00	0.00	0.00	0.00	0.00	0.00	0.00	0.12	0.00	0.00	0.18
55-59	Female	0.00	0.00	0.00	0.00	0.00	0.00	0.00	0.00	0.00	0.00	0.00	0.00
	Male	0.00	2.63	0.00	0.00	0.00	0.00	0.00	0.00	1.45	0.00	0.00	0.62
	Both	0.00	1.30	0.00	0.00	0.00	0.00	0.00	0.00	0.72	0.00	0.00	0.31
60-64	Female	0.00	0.00	0.00	0.00	0.00	0.00	0.00	0.00	0.00	0.00	0.00	0.00
	Male	1.32	3.28	0.00	0.00	0.00	0.00	0.00	0.00	0.36	0.00	0.00	0.45
	Both	0.64	1.59	0.00	0.00	0.00	0.00	0.00	0.00	0.18	0.00	0.00	0.22
65-69	Female	0.00	0.00	0.00	0.00	0.00	0.00	0.00	0.00	0.00	0.00	0.00	0.00
	Male	0.00	0.00	0.00	0.00	0.00	0.00	0.00	0.00	0.51	0.00	0.00	0.16
	Both	0.00	0.00	0.00	0.00	0.00	0.00	0.00	0.00	0.25	0.00	0.00	0.08
70-74	Female	0.00	0.00	0.00	0.00	0.00	0.00	0.00	0.00	0.00	0.00	0.00	0.00
	Male	0.00	0.00	0.00	0.00	0.00	0.00	0.00	0.00	0.00	0.00	0.00	0.00
	Both	0.00	0.00	0.00	0.00	0.00	0.00	0.00	0.00	0.00	0.00	0.00	0.00
75-79	Female	0.00	0.00	0.00	0.00	0.00	0.00	0.00	0.00	0.00	0.00	0.00	0.00
	Male	0.00	0.00	0.00	0.00	0.00	0.00	0.00	0.00	0.00	0.00	0.00	0.00
	Both	0.00	0.00	0.00	0.00	0.00	0.00	0.00	0.00	0.00	0.00	0.00	0.00
80 Plus	Female	0.00	0.00	0.00	0.00	0.00	0.00	0.00	0.00	0.00	0.00	0.00	0.00
	Male	0.00	0.00	0.00	0.00	0.00	0.00	0.00	0.00	0.00	0.00	0.00	0.00
	Both	0.00	0.00	0.00	0.00	0.00	0.00	0.00	0.00	0.00	0.00	0.00	0.00
All Ages	Female	0.05	0.00	0.00	0.00	0.00	0.08	0.00	0.00	0.07	0.00	0.00	0.03
	Male	0.88	0.86	0.11	0.19	0.03	0.20	0.18	0.15	0.43	0.06	0.00	0.31
	Both	0.47	0.43	0.06	0.09	0.01	0.14	0.09	0.08	0.25	0.03	0.00	0.17

Supplementary Figure 1: Surviving (left) and non-surviving (right) alcohol-poisoned cases per 100,000 inhabitants during COVID-19 pandemic in 11 provinces of Iran (n=795)
